# Shared experiences, shared support: A qualitative study on the importance of relatability in interpersonal relationships for youth mental health in South Africa

**DOI:** 10.1017/gmh.2024.117

**Published:** 2024-11-18

**Authors:** Junita Henry, Fredric Azariah, Matt Hughsam, Sarah Skeen, Mark Tomlinson, Chuma Busakhwe, Khotso Mokoena, Almaaz Mudaly, Moitreyee Sinha, Christina Laurenzi

**Affiliations:** 1 Institute for Life Course Health Research, Faculty of Medicine and Health Sciences, Stellenbosch University, Tygerberg, South Africa; 2 Harvard T. H. Chan School of Public Health, Department of Global Health & Population, Harvard University, Cambridge, USA; 3 citiesRISE, Chennai, India; 4 citiesRISE, New York, USA; 5 School of Nursing and Midwifery, Queens University Belfast, Belfast, UK

**Keywords:** youth mental health, relationships, relatability, evidence-based, engagement, South Africa

## Abstract

Adolescence is a critical developmental period marked by significant changes, increasing the risk of mental health problems such as anxiety and depression. Understanding how youth engage with mental health resources is essential. This study explored the role of interpersonal relationships—including peer-to-peer, adult-youth, parent, teacher and mentor relationships, and interactions with mental health professionals—in shaping youth mental health engagement and identified factors influencing these relationships. Using a phenomenological qualitative design, youth researchers (YRs) and youth advisors (YAs) were engaged throughout the research process. Semi-structured interviews were conducted with South African youth aged 14–24 years. The study highlighted the significance of peer relationships, particularly relatability, as key in youth mental health support. Family relationships had a mixed role, with factors like lack of mental health literacy, age differences, and cultural norms hindering effective communication and support. By understanding the dynamics of these relationships, this study emphasizes the need for targeted interventions that harness social support. Enhancing the quality of relationships and promoting positive social bonds can protect against mental health problems. Addressing gaps in support by recognizing and supporting peer-to-peer engagement is essential. Findings provide valuable insights for designing strategies to promote mental well-being among youth, particularly in resource-constrained settings.

## Impact Statement

This study highlights the critical importance of interpersonal relationships in supporting youth mental health, particularly emphasizing peer relationships. By uncovering the dynamics of peer-to-peer support, the research provides actionable insights for developing mental health interventions that leverage these natural support systems. The study’s findings call for improved mental health literacy to bridge generational gaps and enhance communication. Additionally, it stresses the necessity of fostering trust and relatability in mental health support networks. These insights are important for designing effective mental health strategies in resource-constrained settings.

- Relationships play a vital role in promoting youth mental health.
- Peer relationships are critical sources of mental health support for youth.
- Familial relationships play a mixed role in support-seeking.
- Mental health literacy is key to reducing intergenerational gaps in support-seeking.
- Relatability and trust are essential characteristics that facilitate support-seeking.
- There is a disconnect between science and “experience-based” information.

## Introduction

Adolescence is a critical developmental period marked by rapid physical, social and psychological changes (Vijayakumar *et al.* 2018). During this time, adolescents face an increased likelihood of experiencing mental health issues such as anxiety and depression. The majority of mental health disorders (62.5%) emerge by age 25 (Solmi *et al.* 2022), shaping the formation of healthy relationships, goal-setting abilities and decision-making skills (Bentivegna *et al.* 2022; Copeland *et al.* 2015; Ford *et al.* 2023; Kessler *et al.* 2007).

In Sub-Saharan Africa, the prevalence of mental health disorders among adolescents is significant, with studies indicating that up to 20% of adolescents suffer from conditions such as depression and anxiety (Cortina 2012). The region faces unique challenges, including limited access to mental health services, cultural stigma and socio-economic hardships that exacerbate these conditions. These issues are particularly acute in low- and middle-income countries (LMICs), where adolescents and young people (referred to as youth) often face additional stressors linked to poverty, inequality and political and environmental crises, such as conflict and natural disasters (Rose-Clark et al. 2024).

In South Africa, these stressors are compounded by specific social and structural challenges, such as high rates of interpersonal violence, substance use and unemployment—that can exacerbate poor mental health (Human Sciences Research Council 2018; Kempen 2014; Khuzwayo *et al.* 2018; Mokitimi *et al.* 2019; Pantelic *et al.* 2020; Statistics South Africa 2021). For instance, the unemployment rate for youth aged 15–24 is 49%, and the country faces one of the highest rates of interpersonal violence globally, with an average of 21,000 murders reported annually (Crime Statistics South Africa 2022; Statistics South Africa 2021). Recent data indicate that approximately one in five South African adolescents experience some form of mental health disorder, with depression and anxiety being the most prevalent (South African Depression and Anxiety Group 2017). These challenges are aggravated by inadequate mental health policies and fragmented, inaccessible services (Nicholson 2008; SA Federation for Mental Health 2018; Vergunst 2018).

Given these challenges, it is critical to understand how and where youth access information about, and support for, mental health. A UNICEF study conducted in South Africa with 18- to 25-year-olds found that friends, the internet, and, to a lesser extent, family and school, were the main sources of information and support for youth (Tshuma 2017). However, youth also critiqued this support, questioning the reliability of information from friends, and seeing some parental advice as “too traditional.” The study emphasized the importance of friends as a source of social support, primarily through platforms like WhatsApp (Tshuma 2017). Additionally, while youth value accurate information from health workers, they may avoid these sources due to unfriendliness, judgment, and lack of confidentiality (Field *et al.* 2020; Tshuma 2017). This indicates a gap in relatable and trustworthy sources of support, emphasizing the importance of understanding the dynamics of interpersonal relationships in mental health support.

Health service support and behavioral interventions can promote positive mental health and prevent disorders, helping youth navigate academic pressure, social anxiety, and relationship difficulties (González and Macho-Stadler 2013; Huang *et al.* 2007; Laurenzi *et al.* 2021; Skeen *et al.* 2019). Additionally, social support can protect against the development of mental health problems, promote positive psychological well-being (Beeble *et al.* 2009; Fritz *et al.* 2018; Harandi *et al.* 2017), and influence support-seeking behavior (Graber *et al.* 2016). However, there is limited knowledge about what youth value or prioritize in seeking information or support for mental health, especially among younger adolescents defined in this paper as those aged 10–14 years (World Health Organization 2017). Leveraging mechanisms that resonate with youth can enhance health promotion linked to mental health.

### The role of relationships

The role of relationships is particularly significant in this context. Relationships with parents, siblings, friends and romantic partners may provide support and stability during difficult times, offering a sense of belonging and bolstering positive self-image development (Wang *et al.* 2018). Within relationships, youth learn crucial social skills, cooperation and competition, shaping their understanding of interpersonal dynamics (Bukowski *et al.* 2020). Strong social support systems are linked to better mental health outcomes among youth, with supportive friendships associated with lower rates of depression and anxiety (Gariépy *et al.* 2016) and improved resilience (Graber *et al.*, 2016). Conversely, relationships can be a source of stress or conflict. Less healthy relationships—characterized by conflict or engagement in risky behaviors such as substance use—can contribute to poorer mental health (Dishion *et al.* 1999; Steinberg *et al.* 1989). Thus, understanding how to enable adolescents to form positive relationships and what drives these relationships is critical (Moore *et al.* 2018; Sieving *et al.* 2017). By integrating the concept of relatability into this discussion, we can better understand how youth choose and trust the sources of their mental health support.

In South Africa, where formalized mental health services are often limited, early relationships and social support play a role in promoting mental health and preventing mental health disorders. This paper broadly refers to social support as both instrumental (advice giving) and social-emotional support (warmth and care; Cohen and Wills 1985). Understanding which relationships are critical to youth well-being, and what aspects of these relationships are most important can guide interventions aimed at improving mental health engagement in South African families, communities and broader youth ecosystems.

Therefore, despite the growing recognition of the importance of mental health support for adolescents, significant gaps remain in our understanding of what youth value in such support. Specifically, there is limited knowledge about the role of relatability in determining the effectiveness of mental health support systems and what factors influence youth preferences for different sources of support. Additionally, the unique challenges faced by South African youth, such as high rates of violence, unemployment and inadequate mental health services, highlight the need for targeted interventions that address these specific contexts. By addressing these gaps, this study aims to contribute to a more nuanced understanding of youth mental health support, with the ultimate goal of improving outcomes for adolescents in South Africa.

## Study aim and partner

As part of a multinational study on youth mental health information and support conducted across India, Kenya, Rwanda and South Africa, this study explored the role of relationships in shaping youth mental health engagement in South Africa. Our study used a phenomenological qualitative design to explore (1) the relationships that youth identified as crucial in their process of seeking mental health support; and (2) to address the research gap and gain insights into the factors influencing these relationships. The larger project was led by citiesRISE, a multi-stakeholder initiative founded in 2017, committed to transdisciplinary work that aims to address key challenges and opportunities in youth mental health, driving paradigm shifts in its conceptualization and treatment.

## Methods

Ethical approval was granted by Stellenbosch University’s Health Research Ethics Committee (N21/07/062).

### Youth participation

We engaged youth at all levels of the research, from study inception to dissemination of findings. This approach responded to a gap in meaningful participatory involvement of youth across the life cycle of research projects (Rouncefield-Swales *et al.* 2021).

Youth participated as Youth Researchers (YRs, n = 3) and Youth Advisors (YAs, n = 8). Youth residing in South Africa between ages 14–29 years were eligible to join the project as YRs or YAs. YRs were recruited using a purposive sampling technique, where three YRs, aged 23–27 years (JH, KM and CB), were recruited *via* advertisement and word-of-mouth to ensure they had the necessary interest and commitment to the project and were involved in all project steps. This prolonged engagement allowed YRs to build trust with participants and gain a deep understanding of the context, which increased the credibility of the findings.

YRs collaborated with adult researchers to develop interview guides, collect and analyze data, make sense of emergent findings and code data, and develop dissemination reports. YAs, on the other hand, were recruited using a convenience sampling method through existing youth networks.

Eight additional YAs, aged 15–29 years (including AM), were identified through existing youth networks and engaged at three distinct stages to offer strategic guidance, inform the interview guide and interpret findings. YAs participated in Global Advisory Group meetings, leveraging their knowledge and lived experiences to provide feedback on study findings, but were not directly involved in data collection or coding. YRs and YAs represented diverse ages, racial backgrounds, and geographic regions and were compensated hourly for their time. Table S3 in the supplementary material provides a breakdown of their demographics.

YRs underwent certified human subjects training to ensure familiarity with ethical principles of research and relevant local child protection laws and procedures. YRs also received in-depth training on administering informed consent, data collection practices and referral protocols for participants requiring additional support.

### Recruitment

Eligible participants, youth aged 14–24 years living in South Africa, were recruited through YR and YA networks. YAs and YRs messaged or called potential participants via WhatsApp or phone calls to explain study aims and discuss confidentiality measures.

At recruitment, participants indicated their preference for either a small focus group discussion (FGD) or individual interviews, both designed to be virtual. We purposefully recruited youth from across the mental health spectrum; interested participants self-reported their current mental health status as “well” (not having any mental health problems), “struggles” (experience of mental health problems), or “illness” (having ever received a formal diagnosis). Additionally, we tried to ensure a diverse representation of youth in terms of age, gender, geographic location and racial background. This diversity increases the potential transferability of the findings to other youth populations in similar socio-cultural contexts. Informed consent forms were sent *via* email or WhatsApp, and participants were given multiple opportunities to ask questions before signing. Participants signed and returned forms via email or WhatsApp, after which an interview was scheduled. In cases where participants were under 18 years old, we obtained caregiver consent and participant assent in parallel. At the start of each interview, the consent form was reviewed, and participants could ask questions, clarify details or revoke consent. All information and forms were stored on a password-protected platform.

### Data collection

The semi-structured interview and FGD guide were drafted by the core research team for all countries and adapted for the South African contexts with YRs. A second round of modifications followed after feedback from three pilot interviews with youth participants, which included simplifying language, and reducing redundancy. Questions aimed to differentiate between actors (peers, family, teachers and health professionals) and channels (pathways or platforms, *e.g.* social media and school), and between seeking factual information on mental health as well as mental health support. Open-ended questions explored with whom, from where, how, and why adolescents engage with sources of mental health information and support. The guides were initially developed in English and subsequently translated into isiXhosa to ensure accessibility for participants who preferred to be interviewed in isiXhosa.

We conducted virtual, semi-structured interviews and FGDs with youth via Zoom or WhatsApp calls in November–December 2021. To protect confidentiality, interviewers emphasized the importance of privacy and confidentiality at the start of each session. Participants were encouraged to choose a time and setting where they felt comfortable and secure in sharing their experiences. YRs were able to conduct interviews in English or isiXhosa, although not all participants could be interviewed in their first language. Interviews conducted in isiXhosa were audio-recorded and subsequently translated and transcribed simultaneously into English. While best practices typically recommend 4–8 participants per focus group, we conducted FGDs in smaller groups of trios or pairs. This approach was based on participant preference and the sensitive nature of the topics discussed, allowing for a more intimate setting where participants felt more comfortable sharing their experiences. Interviews were audio-recorded with consent, averaging an hour. At the end of each interview, participants were sent a virtual R180 grocery voucher (approximately US$12 at the time) for participation, commensurate with local protocols for participant reimbursement. Interviews were manually transcribed verbatim from audio recordings by an experienced transcription team; interviews conducted in isiXhosa were translated and transcribed simultaneously. Data, including audio recordings, were stored on password-protected devices only accessible by the research team. Transcriptions excluded personal identifiers to ensure anonymity and were quality-checked. We maintained a detailed audit trail throughout the research process, documenting all decisions, changes to interview guides, and steps taken during data collection and analysis. Reflexive memos were kept to document these reflections, ensuring that the findings were grounded in the data rather than influenced by researchers’ biases.

### Data analysis

Data were analyzed by YRs, using thematic analysis (Braun and Clarke 2006) across six stages: familiarization with data, coding, searching for themes, reviewing themes, defining and naming themes and writing. Data familiarization was iterative, entailing audit log-keeping, data collection and preliminary analysis. Building on *a priori* codes derived from the study aims, conceptual framework and interview guide questions, inductive codes were derived by examining finalized transcripts. Initial coding in Dedoose software was completed by YRs, with each interview as the unit of analysis. Parent codes reflected broader themes (*e.g.*, the characteristics of actors and channels), while child codes captured specific characteristics and features of the parent code. Saturation was determined based on code frequency and meaning (Hennink and Kaiser 2022).

To ensure coding quality and rigor, each coded transcript was re-read by either a YR or YA. Discrepancies in coding were discussed and resolved collaboratively, leading to adjustments in the coding framework as necessary. This iterative coding process contributed to the reliability and dependability of the analysis.

To enhance the credibility of our study, we used triangulation by validating findings across multiple sources, including participants, stakeholders and YAs (Tracy 2010). Preliminary findings were shared with YAs to verify the accuracy of interpretations. We adhered to the Consolidated Criteria for Reporting Qualitative Research (COREQ) guidelines (Tong *et al.* 2007).

## Results

### Sample

We engaged 27 youth aged 14–24 years (Table 1). Of the potential participants approached (*n* = 38), 11 did not respond. Interviews were conducted individually with 20 participants, with 7 FGD participants. The majority of interviews were conducted in English; two interviews used a combination of English and isiXhosa. The sample comprised adolescents from four South African provinces (Western Cape, Gauteng, KwaZulu-Natal, and Free State), with the majority identifying as Black/African (74%). Over half of the participants (52%) self-identified as having mental health struggles and/or illness. Table 1 summarizes participant characteristics.^1^

**Table 1.** Sample characteristics

Characteristics		*n*	Mean, SD/ %
Mean age in years		27	18.6, 3.05
Gender	Female	13	48%
	Male	14	52%
Activity status	Employed	2	7%
	University student	10	37%
	High school student	13	48%
	Unemployed	12	44%
Province	Gauteng	12	44%
	Western Cape	6	22%
	KwaZulu-Natal	7	26%
	Free State	2	7%
Race	Black/African	20	74%
	Colored*	3	11%
	Indian	2	7%
	White	2	7%
Mental health status	Well	13	48%
	Struggles	11	41%
	Illness	3	11%
Interview type	Individual	20	74%
	Pair/Trio	7	26%

* This term is broadly used in South Africa to refer to individuals of mixed race and ethnicity, descendants of African, European and Asian people.

Results are organized according to the main emerging themes. Saturation was achieved at around 10 interviews, for 90% of codes and 16–24 interviews for meaning saturation.

#### 1. Diverse relationships shape mental health

Youth described various relationships shaping their engagement with mental health support, including informal peer-to-peer relationships; adult-youth relationships; and interactions with formal sources of support, such as mental health professionals. Participants mentioned key individuals from whom they sought support, while also noting tensions with certain relationships that provided support while sometimes contributing to mental health challenges.

### “Friends are maybe a better family”: Peer relationships as critical but tenuous

Youth described their friends as providing easily accessible support and depth of relationship and intimacy that they could rely on in times of need*: “Normally when I have stress, I normally talk it out with my best friend and other friends…just to sort out each other’s problems”* (Participant ID: CR-11, Age: 16, Self-identified gender: F). Friends were often the “first response,” when youth faced uncertainty about where else to turn with questions or concerns. Some participants preferred peer relationships over those with parents or mental health professionals.

*“I feel like if someone is struggling with mental health, I feel like they would somehow talk about it with their friends. I feel like that is the best thing they can do because friends are maybe a better family because I feel like…mental health is not promoted as much and…we as like, the youth, do not really know where to go if you actually suffer from mental health, you do not even know how to approach it”* (CR-14, 16-M).

*“Telling your problems to your peers is much easier than telling your problems to your parents”* (CR-24, 15-F).

However, issues of trust and judgment shaped peer-to-peer engagement. As youth navigated shifting friendships, opening up to friends was essential but potentially risky, necessitating careful evaluation.

*“[Friends] are normally difficult to find because now in this generation you can’t actually find true friends because they always backstabbing you somehow.”* (CR-7, 16-F).

*“There will always be this thing that “I’m going to be judged, so let me not share with my peers”* (CR-21, 14-F).

“*I am not sure if I know so much about confidentiality, but, it’s not like friends are oblivious and don’t know what trust is, so I think it depends on the people you spend time with or the people you ask for help from*” (CR-22, 16-F).

### “It’s a matter of comfort”: Intergenerational relationships and distinct understandings of mental health

Participants spoke about relationships with parents and family members within their cultural contexts, noting intergenerational and often culturally embedded differences in how mental health was understood. In general, younger generations were described as more comfortable discussing mental health, with mixed feelings about what older family members could contribute. Some participants spoke about the role of culture in shaping their understanding of mental health. As one participant reflected:

*“It’s a matter of comfort. Most black parents don’t create that comfortable space as to say ‘you can talk to me about anything and everything’ because even if you do talk, they will consider you as being dramatic or just overreacting…So, people feel that talking to their close ones will make [their family] judge them even more, because some of those matters include exactly what they are doing to them.”* (CR-1, 21-F).

Other participants felt they could connect with elders. Speaking of his father’s valuable contribution, one participant said: *“I look up to him, I am the person because of him…life is too short, if I don’t speak to him who else can I speak to? He is there for me, I am there for him”* (CR-19, 23-M).

In other cases, older family members might be supportive, but not the preferred first choice to discuss mental health:

*“My older family members would not be as keen on it, but they wouldn’t be like, shut you out, they will be that open…I do feel it is a lot easier for me to converse with these things with my peers and my younger family members than I do with like older people”* (CR-20, 21-M).

For some, sharing struggles with parents added complexity. Some participants described these relationships as long-term and worried about burdening their parents, meaning that mental health issues had to be broached more cautiously.

*“You tell your parents that you are not doing so well, you might feel like you are burdening them or like you are doing something that could change the way that they see you or care about you, but since they are so important, that will really affect you. But with friends, they will come and go, so it’s not as impacting”* (CR-24, 15-F).

Participants also spoke about managing intense emotions with parents, including feeling rejected or misunderstood. The idea of “deep” effects was echoed by several participants:

*“Because they are a lot closer to you…their reactions will cut a lot deeper”* (CR-20, 21-M).

*“Like with your parents, it becomes extremely personal and it’s like…I don’t know how to explain it, but it becomes very deep”* (CR-24, 15-F).

*“Some lack support from their own homes to discuss mental health, whereas others they have a very, um, open platform to discuss everything and anything, but because they want to be seen as the better person, they would rather not.”* (CR-10, 16-F).

Other perspectives characterized this disconnect as simply part of being a young person and learning how to be vulnerable, even with those who are supposed to be close to you:

*“Just because someone is…afraid to share [with their parents]…that doesn’t mean that their parents did a bad job. I think it’s such a scary concept of sharing your weakest point with someone else”* (CR-21, 14-F).

### “Our parents don’t give us a chance”: Parents as the source of pressure

Linked with perspectives on intergenerational relationships, participants also described parental pressure to perform academically or pursue a different path than what they wanted for themselves. As one participant noted, *“I believe our parents don’t give us a chance”* (CR-15, 19-M). Misunderstandings could also set foundations for poor mental health:

*“It’s a different generation now, and I feel like some parents don’t like try and understand their kids…The kids feel pressured in a way which leads them [to] feel depression. Could be that…they wanna pursue a different career, but their parents want them to be doctors, lawyers, scientists”* (CR-17, 21-F).

Some participants described parental pressure emerging with the transition to university life—especially for individuals who came from families where tertiary education was not possible for their parents because of socioeconomic or racial exclusion during apartheid. In some cases, expectations were also internalized, amplifying participants’ isolation:

*“When you [are] in school and you [are] struggling you can’t really tell anyone because like everybody is…expecting you to be a schoolkid and do what is expected of you right? But it gets too much, like academic skills get very overwhelming and you have no one to talk to…it just becomes worse.… There [is] so much expectation and they can’t do it right, and also because our parents are not expecting failure. Like our parents always want us to perform 100% and so you [are] so scared of disappointing your parents…it ends up affecting our mental health, right?”* (CR-02, 19-F).

### The varied landscape of professional support

Youth experiences with mental health professionals varied. Numerous participants recognized psychologists and social workers as available in their schools, universities, or local clinics. However, access to professionals may depend on socioeconomic status and resources. For those with access, connecting with mental health professionals was easy, but accessibility varied by setting and individual initiative.

*“For people who have access to the internet and have access to schools that have psychologists, it is easily accessible, and I also found that there are a lot of apps, like therapy apps where you connect with…they call it a listener, where you can just, where you don’t expect anyone to do anything but just to speak to people to get stuff off your chest”* (CR-22, 16-F).

As another participant shared, the face-to-face aspect of seeing a psychologist, embedded within the university, was encouraging and felt more reliable: *“For reliable information, I trust the psychologist because I am actually seeing them face-to-face and it’s more reliable because it’s [a] primary source like from the university”* (CR-24, 15-F).

Sometimes, participants mentioned avoiding mental health professionals because of cultural barriers or their parents’ perceptions of mental health:

*“It’s kind of embarrassing for most people [to visit a psychologist]. Secondly…in black society, it’s considered being full of yourself or thinking highly of yourself, it is unusual. It’s not a common thing to do and for most people it’s expensive, they can’t afford [it].”* (CR-01, 21-F).

*“I’m still in therapy sessions, still trying to figure out exactly where this comes from, I think I’ve had it a long time ago, even when I was still in high school. It’s just that when I am at home in the rural areas, things like this, it will be like no it’s just me…even my mother…[won’t] really pay attention unless I am coughing or when it is something physical that they can see and take me to hospital. This is [a] simple thing that I used to say this is not so much to think about but when I came here [to the city], I realized that this is serious.”* (CR-12, 23-M).

While participants acknowledged mental health professionals as knowledgeable, they were not always relatable. One participant reflected on the limitations of professionals and the need for more relatable, youth-friendly spaces. She described supporting other students within her university residence:

*“I think we need to have like more support groups, like besides therapists and counselors because sometimes we don’t want to talk with a therapist or a counselor…for example, a therapist that I once went to, she just told me, ‘there’s nothing you can do, it’s all in your brain,’ she just didn’t help in a way, and I will ask a good therapist out there. I think we just need support groups like around our own age groups where everybody has more or less the same issues”* (CR-05, 15-F).

## 2. Trust and relatability as key factors for relational engagement in mental health support

**Figure 1.**
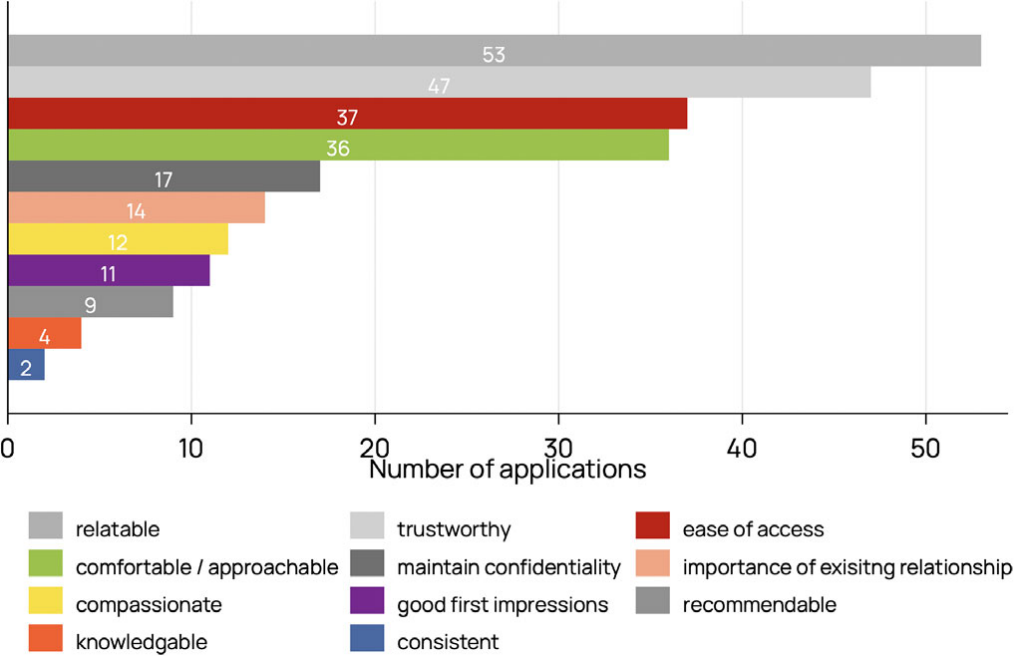
Characteristics of actors/channels that youth seek in relationships.

Adolescent engagement with mental health support was characterized as diverse and influenced by personal experience, social and cultural context, and accessibility. Relatability and alignment, trust, comfort and ease of access were key drivers. Figure 1 shows the prevalence of codes used to describe youth preferences in engaging with mental health support.

### Trust is key and bi-directional

Trust emerged as a key concept that shaped and characterized participants’ journeys engaging with mental health. Trust is also related to characteristics such as the source of support being relatable, impartial, confidential, honest, and easily available (especially for in-person or face-to-face contact) when needed.

Trust was seen in two forms. The first was that the adolescent was comfortable enough to share the entirety of the information. One participant puts it *“If I don’t give them the full picture, they could be recommending something that won’t be as useful”* (CR-21, 14-F). Second was the certainty that the information or support provided by the actor is truthful, credible, and correct: *“I have to trust that what they are telling me will not work against me.”* (CR-21, 14-F). Participants expressed that finding actors to trust was complicated, especially if the trust was violated previously or placed in misleading individuals.

### Speaking to those who can relate

Participants preferred individuals who could understand, empathize with, and relate to their struggles—seeking connections with peers who had similar backgrounds, or shared experiences or challenges. This relatability provided a sense of validation and comfort, allowing them to open up and seek support without fear of judgment or misunderstanding. One participant simply described it as: *“What makes me go to my friend is because he is the only person who really understand[s] what I’ve been through, like he understands me for just being me.”* (CR-07, 16-F).

Relatability also led to the uptake and acceptance of solutions and diverse strategies.

*“[If] the person had the same issue that I have and the person was able to deal with it in a different way and that way helped with them, chances are it’s gonna be easier for me to also use their solutions or the methods that they used then get better”* (CR-01, 21-F).

Trusting relationships also enabled participants to more easily share the totality of their experiences and gain more relatable information—allowing them to fully express themselves. Gendered understandings of trust emerged, too. The notion that *“men don’t cry”* came up frequently, and one participant described, *“males especially, they do not like to speak about how they feel because some people have been brought up that way, they feel if they express how they feel it’s a sign of weakness”* (CR 18, 22-M). Participants also expressed that females were likelier to be open with friends than males: “*what I have noticed with girls, they speak to their friends…But we as guys we do not usually talk.”* (CR-12, 23-M).

### The appeal of “experience-based” information

Entwined with relatability was the tension between evidence and experience. While participants saw the benefit of evidence- and science-based approaches, they also shared reasons for preferring “experience.” They perceived science as lacking the capacity to embrace youth vulnerability. Customizing scientific knowledge to fit individual contexts or situations was deemed more challenging. Consequently, participants viewed firsthand experience or “experience-based” support as better tailored to personal circumstances compared to less relatable scientific approaches. With low existing levels of mental health literacy, introducing scientific concepts was perceived as overwhelming.

Other participants also elevated “experience” over evidence: highlighting the role of friends, peers, or others who might understand what they were experiencing, based on their prior experiences. Sometimes this dovetailed with science-based approaches, but often it was more subjective and personalized. As one participant explained:

“*I feel like when [youth] see something that is experienced by another person, then they would be like this is much easier to go about and this is something I experienced, it is something I know of, and something I am going through, and you can actually talk about it more.”* (CR-14, 16-M).

Some participants felt they could learn about possible solutions from those with lived experience:

*“[If] you need help with a certain situation, it would be better to seek it from someone who has experienced it, like I said, because that person is the one that can give you the perfect, um, solutions to your problems”* (CR-04, 20-F).

In other cases, participants reflected more critically on what shared experiences might facilitate. One participant spoke about different solutions working for different people, and noted that understanding the resources and journey may be instrumental for people:

*“People educating other people about their personal experiences, I often find it very powerful when someone has gone through the same thing that I went through when I feel stuck, and they give resources that they used to help them. But again, not everything is the same for everyone, but just I find that personal experience and education about personal experience helps people.”* (CR-22, 16-F).

This participant further spoke about seeing living proof in that individual, the “person themselves” as evidence that the solution may be effective. In some cases, personal experiences were explicitly “non”-evidence-based:

*“They do not give me evidence-based information, they give me information that is, um, experienced, so…something that they have noticed or they have heard of.”* (CR-14, 16-M).

Fact-checking was still part of accepting this support: as one participant stated, *“In general, there’s no science really involved, it’s just personal connections and talking”*, but assessing credibility was possible, because *“sometimes if it doesn’t add up,”* you know it may not be credible (CR-20, 21-M).

Other participants reflected on how culture, and “traditional” approaches, were sometimes seen to conflict with Westernized conceptualizations of mental health and wellbeing:

*“First of all culture, it should be so it should not be a matter of…culture and science. Science should not be treated as superior to culture…They should be treated as equal, one should not be bigger than the other, because when one is bigger than the other, then obviously the other one will be, um, I do not want to say downgraded or degraded. I do not know how to call it, but one will be undermined, as if it is bad over the other, so I think they must be treated equally”* (CR-03, 24-M).

For some respondents, evidence-based approaches were recognized as important in expanding psychoeducation and lending credibility to mental health support. One participant noted, *“Youth need to be informed well about all these mental illnesses, because I feel like most of them done self-diagnose and they do it wrong, you see”* (CR-02, 19-F).

For others, embracing evidence-based practices worked for them—but was not necessarily a blanket approach to recommend for all youth. *“When it comes to mental health, as much as we can put in science there, it is important for us to put science there, but then I think everybody is different”* (CR-03, 24-M).

## Discussion

This study explored the relationships that South African youth identified as critical in their engagement with and promotion of mental health and the factors that shaped these relationships.

Personal relationships, particularly peer-to-peer engagement, were identified as crucial but fragile sources of mental health support. Youth valued the relatability and understanding that peers could provide, with trust being central. Female participants were likelier to discuss their mental health within their peer groups compared to their male counterparts. Our findings align with the broader project and existing literature, which also emphasize the significance of peer relationships in youth mental health support (CitiesRise 2022; Tshuma 2017). We did not find evidence that youth trusted more “effective” strategies. However, trust may change with the experience of lived mental health issues, enabling youth to better identify channels and actors.

To address gaps in current access to support, it is crucial to recognize the significance of peer-to-peer engagement and provide support mechanisms within this context (Butler *et al.* 2022; Roach 2018; UNICEF *et al.* 2021; Williams and Anthony 2015). Trained peer counseling int0065rventions have been identified as valuable modes of mental health support (Simms *et al.* 2022), and mechanisms such as reducing stigma, expanding social connections, practical coping strategies, and developing client autonomy have been identified as core components of effective peer support (Halsall *et al.* 2022). However, more empirical evidence is needed, particularly for younger children and adolescents in LMICs (Simmons *et al.* 2023; Tisdale *et al.* 2021).

Family relationships played distinct, at times conflicting roles in youth mental health support, with culture playing a critical role in shaping these dynamics. While family support can be protective, effective communication and support may be hindered by limited mental health literacy within families, generational differences between parents and youth, and cultural norms. Particularly within Black South African families, traditional cultural values often influenced how mental health issues were understood and addressed, sometimes creating barriers to open communication.

Interventions that address parent-youth communication and enhance mental health literacy within families by targeting multiple generations could increase mental health literacy, foster social interactions, and bridge intergenerational gaps within households.

Our results highlight the disconnect in preferences between science and “experience-based” information. Most participants spoke about the appeal of the latter, emphasizing the need to raise mental health awareness across ages and genders, incorporate mental health literacy into the school curriculum, and deliver information in a digestible, relatable manner. The complexities within family relationships and the influence of cultural norms, curriculum, and intergenerational differences can also be understood within the framework of ecological systems theory (Bronfenbrenner 1996), recognizing youth as embedded in multiple systems, including the family, school, community, and broader societal contexts. Incorporating mental health literacy at both home and school levels may actively enhance positive mental health and well-being for youth.

Our findings point to the importance of considering distinct factors when providing mental health support to adolescents. Factors such as relatability and personal experience are influential, low-hanging fruit that should cut across multiple sectors. A study conducted in two general hospitals in North West province, South Africa, with mental health nurses, revealed the need for psychosocial services to include: school health education, counseling, problem-solving, adolescent-friendly user services in facilities, peer education, and the use of immediate adolescent environments to foster recovery and role modeling (Chukwuere *et al.* 2021). These results relate to health literacy and help-seeking models, emphasizing the need for increased mental health literacy and incorporating mental health education into the curriculum to improve youth engagement with evidence-based information and services (Van Der Westhuizen *et al.* 2023). Given the cultural context, these interventions must be culturally sensitive and tailored to align with the values and beliefs of the communities they serve (Van Der Westhuizen *et al.* 2023).

These findings also highlight the need for creative approaches to make science-based information more appealing and relatable to youth. Entertainment, education, and communication models (Cournos 2004) that emphasize the use of entertainment media to deliver health messages and engage audiences in behavior change may be one option. Researchers and practitioners should further explore factors influencing youth help-seeking behavior and develop interventions that effectively address the identified gaps and preferences.

This study has both strengths and limitations. While efforts were made to include diverse participants, the sample is not fully representative of South Africa’s population. Participants were recruited through existing networks, which could result in self-selection bias. Those who chose to participate might have been particularly interested in mental health, and mental health status was self-reported rather than formally diagnosed. Although we attempted to conduct interviews in preferred languages, language barriers or cultural nuances may have shaped our data. Interviews were conducted virtually through Zoom or a telephone call, limiting non-verbal cues and potentially affecting interviewer-participant rapport. Nonetheless, we feel that our multi-level participatory approach is a notable strength. YRs were involved at all stages, and YAs were consulted at various touchpoints throughout the study. While genuine engagement has been treated as a tick-box (Preston 2007) or tokenistically (Hart 1992), engaging youth in intervention design and collaboration is critical (Collins *et al.* 2024). As far as possible, youth engagement was valued and integrated across distinct stages of the research continuum.

## Conclusion

Our findings provide valuable insights into the diverse nature and relevance of youth relationships for their mental health. The study highlights the need for comprehensive support systems that consider the preferences and needs of adolescents—to be relatable and experience-based. Strengthening peer support networks, improving mental health literacy within families and schools and creatively delivering science-based information in a relatable, accessible manner may be promising approaches to enhance the effectiveness of mental health support for youth in South Africa and similar settings, and holistically promote their well-being.

## Supporting information

Henry et al. supplementary material Henry et al. supplementary material

## Data Availability

The data that support the findings of this study are not publicly available due to restrictions related to privacy and ethical considerations. Participants were assured that their responses would remain confidential and would not be shared outside the research team. As a result, the raw data from the semi-structured interviews and related materials cannot be disclosed. For further information about the study, interested parties can contact the corresponding author.

## Footnotes

1
Supplementary file S2 contains a list of participant characteristics.

## Supplementary material

The supplementary material for this article can be found at http://doi.org/10.1017/gmh.2024.117.
