# Supplementary material for: Shared experiences, shared support: A qualitative study on the importance of relatability in interpersonal relationships for youth mental health in South Africa
Source: Glob Ment Health (Camb). 2024 Nov 18;11:e111. doi: 10.1017/gmh.2024.117 (PMC11588417; doi:10.1017/gmh.2024.117)
Supplement: Henry et al. supplementary material [file S2054425124001171sup001.docx]

**Supplementary files**

**S1:Interview guide**

**Background information to gather:**

- How do you self-identify your gender?
- What is your age (date of birth if possible)?
- What is your educational level (highest education completed)?
- What is your marital status?
- What is your current place of residence?
- Is it urban or rural?
- How do you self-identify your race or ethnicity?
- What language or languages do you speak?
- Can you please provide an alternative contact? Family or other.

- **How do you spend your days?**

Probe: As a student, community member, working for someone else, self-employed, supporting a family in the home, just focusing on taking care of yourself and your own health right now, and so on…

**Introduction:**

Today we are going to talk about mental health and wellbeing.

We are going to focus on your perspectives and impressions. We are also going to ask about ACTORS and CHANNELS. Think of these as the individuals, or pathways, through which you learn about and engage with mental health.

Do you/does anyone have an example of an actor or a channel they can think of? (Probe: if no reply, give an idea e.g. a teacher or a friend).

**Questions and Sub-Questions:**

Let’s get started.

1. **What do you understand by the terms mental health and mental wellbeing?**

1. **How do young people talk about their mental health?** We are interested in hearing about the South African setting specifically and the more local settings you live in/engage in. Cultural understandings of mental health are also relevant here.

- What/who comes to your mind when you hear the term mental health? Are specific words used/avoided?
- What issues do youth need mental health information and/or support with?
- What are 3 most used terms to talk about mental health in your community? Can you elaborate a bit more on their meaning? *(pilot question)*
- What are the different ways young people use to express themselves? Do youth share personal accounts, or prefer to talk about their mental health more generically/indirectly?

1. **What do you think is important for your mental well-being?**

- Probe: self-esteem, confidence, self-motivation, humor, helping others, communication, trust, being open-minded, ways of dealing with stress, nutrition, physical health

1. **What do you think are the main challenges facing young people, and how do you think these challenges influence/shape their understanding of mental health?**

- Probe: family history, parenting issues, adverse events in childhood, stressful life events/experiences, lifestyle factors, social factors such as poverty, discrimination, unemployment, substance use, witchcraft, etc.

1. **Do you have lived experience with mental health challenges?**

- Probe for those with a lived experience: Has your understanding changed following your experience? Why, and in what way?

**Let’s talk a bit more about ACTORS and CHANNELS now.**

1. **Which are the consistent actors and channels that young people go to for mental health information?**

- **How** do young people find/**identify actors** and channels?
- Do you feel that they are **easy to find, or difficult?**
- **Where** do young people **get *most* of their information** from when it comes to mental health – on what platforms?
- **What** are the **key characteristics of consistent actors** and channels? What makes them consistent sources?
- Is the actor/channel you go to for mental health the same as those you go to when facing other challenges/problems?
- Are these consistent sources trustworthy?

1. **Where and how do young people engage with the different actors and channels?**

- What is more important to you, a channel or an actor?
- How **often** do young people interact with specific actors or channels? How often are young people engaging with the same actors across multiple channels?
- How can a young person **access** the platforms and individuals that provide mental health information to them? Freely accessible? Pay for the service? Sponsored? In-person? Virtual?
- How do you think engagement patterns vary among youth (age, gender, ethnicity)? You can think of your friends or of your understanding of wider South African society.
- **Do young people engage differently (such as passive or active engagement) with specific actors (e.g., peer) based on the setting they are in? E.g., peer in a school setting versus a faith setting?**
- What in particular shapes the form of engagement? (Access? appeal?)
- Is there stigma attached to mental health / does this affect where and how you or other young people are willing to engage?

1. **How much do young people trust different actors or channels and why?**

- **What motivates youth to trust some actors/channels, over others?**
- **Who/what helps to establish trust with an actor or channel?**
- Why do youth go back to certain platforms/channels for mental health information/support? What would make youth avoid certain platforms or individuals for mental health information and support?
- How **relatable** are the actors/channels to the youth in specific settings? Is it important that youth identify with/align with these actors/channels in a certain way to build trust?
- How much does "trusting" an actor or channel lead to engaging in behaviors that align with their recommendations?

1. **What are the strengths and weaknesses (and facilitators and barriers to effectiveness) of different actors or channels?**

- Would you **recommend** a friend or a family member to use a specific channel or contact that actor? Why?
- How do youth determine what is effective? (is it personal, or broader-based?)
- **Are any of these channels or actors made for youth/by youth?**
- How accessible are the different actors and channels in your setting?
- Which actors and channels lead to the greatest adoption of health promoting behaviors?
- To what extent are actors/channels **evidence-based?**
- How do you assess if the information provided is credible?
- To what extent do the channels and actors that youth access use credible information?
- Does the use of science by an actor/channel make them more effective or appealing?

1. **What do you feel will make evidence or science-based information on mental health more attractive to you and to other young people? What do you think should be done to promote mental health amongst young people?**

- Probe: Do you have an idea of how is best to encourage youth to engage in accessing such science-based information, and what needs to be done?
- How can science and custom knowledge be integrated to create/convey culturally relatable information for youth in specific setting i.e. rural, disadvantaged communities?

**S2: Participant characteristics**

| **CR- (ID)** | **Age** | **Gender** | **Status** | **Province** | **Race** | **Mental Health Category** |
| --- | --- | --- | --- | --- | --- | --- |
| 01 | 21 | F | University student | Gauteng | Black/African | Illness |
| 02 | 19 | F | University student | Gauteng | Black/African | Struggles |
| 03 | 24 | M | Employed | Western Cape | Black/African | Well |
| 04 | 20 | F | University student | Free State | Black/African | Struggles |
| 05 | 15 | F | High school student | Gauteng | Indian | Struggles |
| 06 | 17 | M | High school student | Gauteng | Colored | Well |
| 07 | 16 | F | High school student | Gauteng | Colored | Struggles |
| 08 | 23 | M | Employed | Kwa-Zulu Natal | Black/African | Well |
| 09 | 20 | M | University student | Gauteng | Black/African | Well |
| 10 | 16 | F | High school student | Gauteng | Black/African | Illness |
| 11 | 17 | M | High school student | Western Cape | Black/African | Struggles |
| 12 | 23 | M | University student | Western Cape | Black/African | Struggles |
| 13 | 19 | F | University student | Kwa-Zulu Natal | Black/African | Struggles |
| 14 | 16 | M | High school student | Kwa-Zulu Natal | Black/African | Well |
| 15 | 19 | M | University student | Kwa-Zulu Natal | Black/African | Well |
| 16 | 21 | F | University student | Kwa-Zulu Natal | Black/African | Well |
| 17 | 21 | F | Unemployed | Kwa-Zulu Natal | Indian | Well |
| 18 | 22 | M | University student | Kwa-Zulu Natal | Black/African | Struggles |
| 19 | 23 | M | Unemployed | Free State | Black/African | Struggles |
| 20 | 21 | M | University student | Gauteng | Colored | Struggles |
| 21 | 14 | F | High school student | Gauteng | White | Well |
| 22 | 16 | F | High school student | Gauteng | White | Illness |
| 23 | 17 | F | High school student | Gauteng | Black/African | Well |
| 24 | 15 | F | High school student | Gauteng | Black/African | Struggles |
| 25 | 16 | M | High school student | Western Cape | Black/African | Well |
| 26 | 16 | M | High school student | Western Cape | Black/African | Well |
| 27 | 14 | M | High school student | Western Cape | Black/African | Well |

**S3: Descriptors of YAs and YRs**

| **Age** | **Gender** | **Province** | **Racial identity** | **Affiliations/current work** |
| --- | --- | --- | --- | --- |
| **Youth Researchers (YRs)** | | | | |
| 27 | F | Gauteng | Indian | Researcher |
| 23 | F | Western Cape | Black/African | Honors student |
| 24 | M | Western Cape | Black/African | Honors student |
| **Youth advisors (YAs)** | | | | |
| 15 | Female | Gauteng | Indian | High school student; climate change activist and a multi-award-winning debater. Member of Youth Council for Global Youth Empowerment Circle |
| 18 | Male | Kwa-Zulu Natal | Black/African | University student |
| 24 | Female | Kwa-Zulu Natal | Indian | Unemployed |
| 27 | Female | Western Cape | Coloured | Master’s in Public Health student and research assistant |
| 20 | Female | Gauteng | Black/African | Youth facilitator in a community empowerment programme for young women |
| 29 | Female | Eastern Cape | Black/African | Program manager at an NGO focusing on youth |
| 26 | Female | Western Cape | Indian | Studying towards a Masters |
| 23 | Male | Kwa-Zulu Natal | Indian | Working for a financial consulting company, video editor for NPOs |
