# Supplementary material for: Shared experiences, shared support: A qualitative study on the importance of relatability in interpersonal relationships for youth mental health in South Africa
Source: Glob Ment Health (Camb). 2024 Nov 18;11:e111. doi: 10.1017/gmh.2024.117 (PMC11588417; doi:10.1017/gmh.2024.117)
Supplement: Peer review history

# Editor report: Recommendation: Shared experiences, shared support: A qualitative study on the importance of relatability in interpersonal relationships for youth mental health in South Africa — R0/PR2

**DOI:** 10.1017/gmh.2024.117.pr2

**Affiliations:** Psychiatry, Makerere University CHS, Uganda

No accompanying comment.

# Editor report: Decision: Shared experiences, shared support: A qualitative study on the importance of relatability in interpersonal relationships for youth mental health in South Africa — R0/PR3

**DOI:** 10.1017/gmh.2024.117.pr3

**Affiliations:** London School of Hygiene & Tropical Medicine, United Kingdom of Great Britain and Northern Ireland

No accompanying comment.

# Editor report: Recommendation: Shared experiences, shared support: A qualitative study on the importance of relatability in interpersonal relationships for youth mental health in South Africa — R1/PR5

**DOI:** 10.1017/gmh.2024.117.pr5

**Affiliations:** Psychiatry, Makerere University CHS, Uganda

Dear Authors,

Congratulations for a well written manuscript!

A minor comment: Please clarify on the type of triangulation that was done ie methodological, data or investigator triangulation?

# Editor report: Decision: Shared experiences, shared support: A qualitative study on the importance of relatability in interpersonal relationships for youth mental health in South Africa — R1/PR6

**DOI:** 10.1017/gmh.2024.117.pr6

**Affiliations:** London School of Hygiene & Tropical Medicine, United Kingdom of Great Britain and Northern Ireland

No accompanying comment.
